# Supplementary material for: Association between food insecurity, ethnicity, and mental health in the UK: An analysis of the Family Resource Survey
Source: PLoS One. 2025 Oct 15;20(10):e0332762. doi: 10.1371/journal.pone.0332762 (PMC12527198; doi:10.1371/journal.pone.0332762)

S1 Fig. Directed Acyclic Graph (DAG) food insecurity and long-term health condition affected by mental health

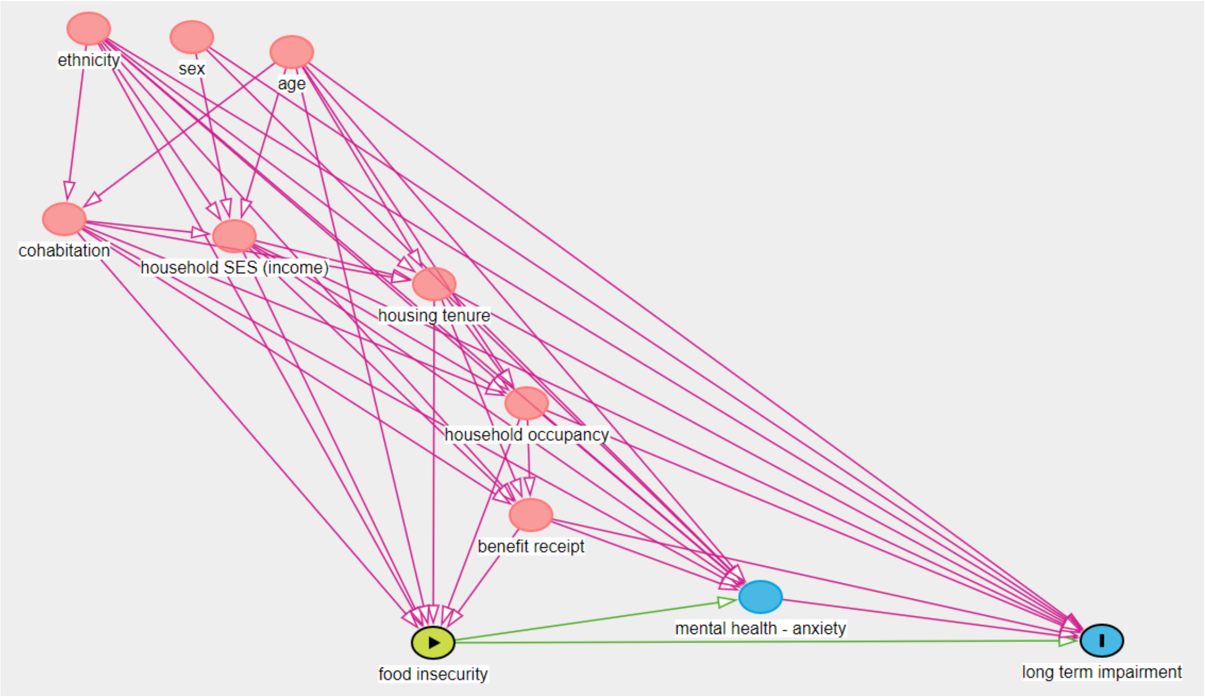

Supplement: S1 Fig — (PDF) [file pone.0332762.s001.pdf]
